# Safety and toxicity assessment of antisense oligonucleotides in brain-relevant human cellular models

**DOI:** 10.1016/j.omtn.2026.103062

**Published:** 2026-08-13

**Authors:** Laura Colar Zanjko, Raúl Andrés-Santamaria, Andrea Balogh, Willeke M.C. van Roon-Mom, András Dinnyés

**Affiliations:** 1 BioTalentum Ltd, Aulich L u 26, 2100 Gödöllő, Hungary; 2 Department of Physiology and Animal Health, Institute of Physiology and Animal Nutrition, Hungarian University of Agriculture and Life Sciences, 2100 Gödöllő, Hungary; 3 Department of Human Genetics, Leiden University Medical Center, 2300RC Leiden, the Netherlands; 4 Central Animal Facility, Leiden University Medical Center, 2300RC Leiden, the Netherlands

**Keywords:** MT: Oligonucleotides: Therapies and Applications, central nervous system, antisense oligonucleotides, *in vitro* toxicology, neurological diseases, personalized therapies

## Abstract

Antisense oligonucleotides (ASOs) have become an established therapeutic modality for central nervous system (CNS) disorders, enabling selective modulation of gene expression through splice-switching or steric-hindrance mechanisms. Backbone and sugar chemistry modifications improved nuclease resistance, tissue retention, and pharmacodynamic durability, facilitating clinical translation of intrathecally delivered ASOs. This review summarizes current *in vitro* approaches for assessing safety and toxicity of CNS-targeted ASOs, alongside the evolving regulatory landscape shaping their preclinical evaluation. Human-relevant platforms—induced pluripotent stem cell (iPSC)-derived neurons, astrocytes, microglia, and brain organoids—enable evaluation of target engagement, cytotoxicity, and immune activation in disease-relevant genetic backgrounds, though each carries distinct trade-offs in throughput, maturity, and translational fidelity. Delivery strategies and cytotoxicity assays, including calcium imaging, lactate dehydrogenase release, metabolic activity measurements, apoptosis markers, and transcriptomic and proteomic profiling, allow detection of sequence- and chemistry-dependent liabilities, including off-target and neuroexcitability effects. Emerging evidence demonstrates sequence and chemical modifications predicting acute neurotoxicity, highlighting the value of systematic *in vitro* screening. As personalized “n-of-1” ASO therapies expand, robust and standardized *in vitro* safety profiling becomes increasingly critical. Comprehensive evaluation in relevant CNS models will be essential to minimize risk and improve translational predictability for developing safe and effective ASO treatments for neurological diseases.

## Graphical abstract

**Figure:**
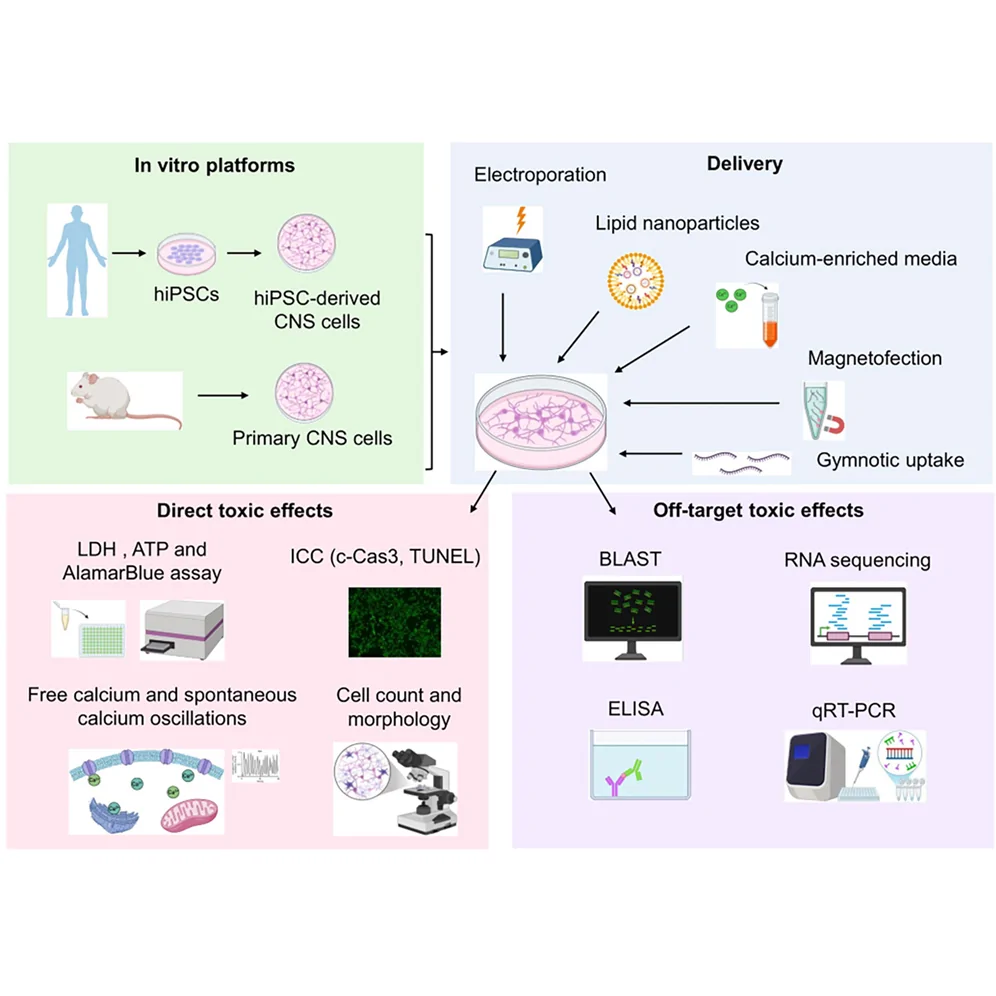

## Teaser

Dinnyés and colleagues reviewed past and current developments in safety and toxicity assessment of antisense oligonucleotides affecting the central nervous system. They provided insights into how their future development can be refined by combining advanced *in vitro* models with state-of-the-art methodology.

## Introduction

Antisense oligonucleotides (ASOs) are short, synthetic, and single-stranded oligonucleotides that can bind to a target RNA through Watson-Crick base pairing. ASOs are highly specific and can alter protein expression by either knocking down mRNA levels, inhibiting translation, or modifying pre-mRNA splicing, ultimately leading to either a reduction, modification, or restoration of a specific protein. The chosen mechanism of action will depend greatly on the disease mechanism. Gapmer ASOs eliminate their target mRNA by hybridizing with it and inducing RNase H-mediated degradation. Apart from RNA degradation, ASOs can act via steric hindrance. Splice-switching ASOs bind to splicing sites on pre-mRNA, modifying the splicing process by skipping or including exons, thus synthetizing a modified, yet functional protein. Translation blockers bind to the translation initiation codon (AUG) to inhibit translation, while maturation/polyadenylation blockers bind to RNA ends and block the addition of the 5′ cap and 3′ poly A tail. Besides pre- or mRNA binding ASOs, there are antimiRs or anti-miRNA ASOs, which bind to miRNAs, blocking the formation of the RNA-induced silencing complex (RISC), enhancing translation. These various mechanisms present ASOs as a highly versatile tool with significant potential for the development of treatments for genetic disorders.^1^ This has resulted in approved drugs for brain disorders such as spinal muscular atrophy (SMA)^2^ and amyotrophic lateral sclerosis (ALS)^3^ and several hyper-individualized ASO treatments.^4^^,^^5^^,^^6^

## Overview of ASO development

The first use of antisense-mediated gene inhibition was published in 1978 by Stephenson and Zamecnik to inhibit Rous sarcoma virus replication.^7^ During the first generation of ASOs, phosphorothioate (PS) backbones were introduced to enhance nuclease resistance, though they increased non-specific protein binding and associated toxicity.^8^ The second-generation ASOs incorporated sugar modifications (e.g., 2-O-methyl (2-O'Me) and 2′-O-methoxyethyl [2′-MOE]) that improved target affinity and stability, allowing for lower dosing and reduced toxicity.^9^ Third-generation ASOs were locked nucleic acids (LNAs), peptide nucleic acids, and phosphorodiamidate morpholino oligomers, which further optimize pharmacokinetic and pharmacodynamic properties, although some modifications (e.g., LNAs) have been linked to increased risk of neurotoxicity as they are prone to chelating Ca^2+^ and Mg^2+^, off-target RNA binding, and RNase H1-mediated cleavage of unintended transcripts.^10^^,^^11^^,^^12^ To offset these effects, chimeric 2′-MOE/LNA wings, mixed phosphodiester (PO)/PS backbones, and site-specific gap modifications (2′-OMe and 5′-cyclopropylene [CP]) can be introduced.^11^^,^^12^ Recent advances like the 2′-O-[2-(methyl-amino)-2-oxoethyl] modification enhance the pharmacological potency of ASOs, permitting lower or less frequent dosing and therefore decreasing toxicity.^13^

ASOs face challenges as systemic delivery leads to high accumulation in the liver, kidney, and spleen but yields very low central nervous system (CNS) exposure because ASOs cannot cross the blood-brain barrier (BBB). Direct delivery methods, like intravitreal (IVT) for the eye and intrathecal (IT) for the brain and spinal cord, bypass the BBB to achieve high local uptake, a long tissue half-life, and low peripheral toxicity.^14^^,^^15^^,^^16^^,^^17^ Despite efficacy, long-term IT delivery poses neurotoxic risks, systemic inflammation, or delayed adverse effects such as hydrocephalus. Because early preclinical efficacy studies often undervalue safety assessments, utilizing *in vitro* toxicity assays is crucial for screening multiple candidates before moving to *in vivo* animal studies.^18^^,^^19^^,^^20^^,^^21^^,^^22^ In humans, two intrathecally delivered ASOs, nusinersen and tofersen, are approved by the Food and Drug Administration (FDA) and the European Medicines Agency (EMA) due to their pharmacodynamic effects modulating SMN2 and SOD1 gene expression.^2^^,^^3^

Individualized, n-of-1 therapies have emerged in the past decade, facing numerous obstacles such as lack of animal models and inability to perform clinical trials.^23^ Therefore, development of computational predictions, *in vitro* efficacy and toxicity assays in patient-derived cells, and *in vivo* safety studies in wild-type animals to assess the efficacy and safety of n-of-1 ASOs before administering them to patients are crucial.^24^ They require a tailored, individualized approach, where long-term risk-benefit analysis is evaluated based on early clinical experience from the specific patient.^6^^,^^25^ Regulatory and clinical frameworks must differ from standard drug development, shifting from large, randomized, and controlled designs to individualized, case-based assessments with flexible endpoints.^23^

## Regulatory framework

Historically broader guidelines such as ICH M3(R2) on non-clinical safety studies for the conduct of human clinical trials and marketing authorization for pharmaceuticals were applied to oligonucleotides.^26^ Regulatory framework has been updated in recent years, with a more focused approach on oligonucleotide therapeutics. Currently under development, the ICH S13 guideline Nonclinical Safety Evaluation of Oligonucleotide-based Therapeutics is focusing on standardizing global regulations for oligonucleotides. Its aim is to establish frameworks addressing the unique chemical, structural, and sequence-dependent risks of oligonucleotide-based therapeutics.^27^

In November 2024, the FDA issued a draft guidance under the title Nonclinical Safety Assessment of Oligonucleotide-Based Therapeutics, wherein they cover criteria for the identification of on- and off-target effects and different types of toxicity studies in non-clinical trials. They mention *in vitro* methodologies as standards for identification of potential off-target hybridization, and following ICH M3(R2) guidelines, they conclude *in vitro* studies present as viable platforms for pharmacological evaluation in the CNS and other vital organs, especially if the drugs are administered locally. An important aspect raised is the relevance of the studies to the class of oligonucleotide, with the specific example of complement activation by 2′-MOE-PS ASOs requiring secondary pharmacology studies (e.g., receptor, ion channel, and enzyme binding and functional screens). There is an emphasis on the 3R principle, thus encouraging exchanging *in vivo* with *in vitro* models when applicable.^28^

The FDA published a separate draft for Nonclinical Testing of Individualized Antisense Oligonucleotide Drug Products for Severely Debilitating or Life-Threatening Diseases. N-of-1 and N-of-few disease therapeutics are not being clinically evaluated and are considered FIH (first-in-human) therapeutics. As there is a greater risk due to limited data, the *in vitro* and/or *in vivo* proof of concept needs to be convincing in meeting certain standards similar to the industrial guides, such as hybridization-dependent off-target assessment, safety pharmacology, and general toxicity. The timeline of the studies can be significantly shortened, and the FDA can provide flexibility in applying regulatory standards because of the individualized approach.^29^

The EMA follows a similar approach, combining their Guideline on the Development and Manufacture of Oligonucleotides with the implementation of ICH guidelines. Similar to the FDA, their focus is on sequence safety and off-target verification, with a strong accent on structural impurities, which can lead to toxic effects.^30^^,^^31^

## *In vitro* models for evaluating CNS-directed ASOs

Since ASOs target human RNA sequences, it is important to develop adequate *in vitro* systems that express the full human transcriptome, preferably in a neuronal context. Platforms such as neuronal, astrocytic, and microglial cultures and co-cultures, brain organoids, and assembloids, derived from healthy individuals and patient-derived induced pluripotent stem cells (iPSCs), reproduce disease conditions in a human genetic background, enabling the assessment of ASO efficacy and safety in a patient-tailored background and enhancing the translation of the ASOs into the clinic. The comparison between these models is available in Tables 1^32^^,^^33^^,^^34^^,^^35^^,^^36^^,^^37^ and 2,^38^^,^^39^.

**Table 1:** 2D *in vitro* platforms utilized for assessment of ASO toxicity

Platform	Toxicity	CNS architecture fidelity	Acute vs. delayed sustainability	Translational limitations
iPSC neurons	loss of calcium oscillations and excitability, synapse destruction, paraspeckle protein mislocalization, and apoptosis	moderate (lack of supporting cells)	excellent acute sustainability (stable culture, high-throughput screening) bad to moderate delayed sustainability (degeneration of synapses)	high reproducibility; lacks physiological tissue architecture and multicellular immune response
iPSC astrocytes	astrogliosis, loss of metabolic support, and dysregulation of glutamate uptake	moderate (lack of supporting cells)	excellent acute sustainability (stable culture, easily adaptable to different standard media) moderate to good delayed sustainability (after several weeks, homeostasis is disrupted and they become reactive)	high reproducibility; lacks physiological tissue architecture and neuronal-glial metabolic interactions
iPSC microglia	sequence-independent inflammation and immunotoxicity	moderate (lack of supporting cells)	good acute sustainability (once differentiated highly reactive) bad delayed sustainability (absence of neurons and astrocytes disrupts homeostasis, inducing activation)	moderate reproducibility; highly varied phenotypes, overly sensitive to stress and immune stimulation

**Table 2:** 3D *in vitro* platforms utilized for assessment of ASO toxicity

Platform	Toxicity	CNS architecture fidelity	Acute vs. delayed sustainability	Translational limitations
Brain organoids	chronic toxicity and tissue distribution	high (lacks vascularization)	bad acute sustainability (requires a long period to differentiate and mature) good to great delayed sustainability (lack of vascularization can lead to core necrosis after several weeks or months)	low reproducibility; highly varied physiology, prolonged maturation, and core necrosis
Assembloids	complex network and circuit disruptions	very high (mimics complex tissues and networks)	bad acute sustainability (requires time for fusion of different regions) great delayed sustainability (allows long-term observation of cell migration and formation of neural circuits over several months)	very low reproducibility; highly varied physiology

Patient-derived iPSC lines are reprogrammed from individuals diagnosed with a specific disease. The cells carry the exact genetic background of a disease, which makes them ideal for the development of personalized medicine and investigating genetically complex diseases with multiple gene mutations. Sourcing them can be difficult, with genetic variability and line-to-line variation presenting problems. Healthy individual-derived iPSC lines are easily sourced, providing a reliable standard for cellular morphology and function, making them great for assessing toxicity. Healthy donors can also have anatomical differences and carry undiagnosed genetic conditions, and the derived cells need to be genetically engineered to replicate complex disease pathologies.

Human iPSC (hiPSC)-derived neurons present as adequate platforms for high-throughput validation, tracking network disruptions, and predicting cell-specific toxicity and off-target effects, while hiPSC-derived astrocytes can track ASO accumulation, metabolic activity, and astrogliosis, identifying cellular stress caused by specific ASO chemistry like PS modifications. hiPSC-derived microglia are very heterogeneous and not as easily scalable as neurons or astrocytes but are great platforms for testing sequence-independent immunotoxicity, such as the activation of Toll-like receptors or the release of pro-inflammatory cytokines, enabling prediction of acute inflammatory responses or injection-site-related neuroinflammation.

Monocultures lack the neuronal-astroglial-microglial interactions, which makes them physiologically less authentic to the human brain. Microglia are highly sensitive to their environment; subsequently, they quickly lose their resting state and exhibit unnatural activation profiles without interaction with neurons and astrocytes. Astrocytes alter neuronal survival and synaptic pruning as a stress response. Neurons are more sensitive without support cells, as astrocytes and microglia influence ASO clearance and inflammation. Creation of co-cultures could mend these drawbacks.

To overcome the disadvantages of simple cultures, incorporation of 3D models can give insight into more complex networks and interactions of ASOs within them. Brain organoids are self-assembled 3D structures generating a complex extracellular matrix (ECM) with diverse cell types (neurons, astrocytes, and sometimes microglia). They offer high predictability of tissue-level pharmacokinetics, spatial distribution, and chronic, long-term exposure toxicity. They can be analyzed with a low throughput and need prolonged maturation. High batch variability and lack of vascularization can lead to necrosis in its core, which can distort toxicity readouts.

By combining specific brain regions, such as cortical and striatal organoids, ASO toxicity can be tracked across specific axonal pathways and neural circuits. These assembloids are structurally extremely complex and can predict circuit- and network-specific neurotoxic effects such as disrupted motor-neuron connectivity. Similar to organoids, they are highly varied, with extremely low throughput, as they are labor intensive.^32^^,^^33^^,^^34^^,^^35^^,^^36^^,^^37^^,^^38^^,^^39^

## *In vitro* delivery methods

Delivering ASOs into cells generally follows similar principles as other nucleic acid-based molecules like small interfering RNAs (siRNAs), miRNAs, and plasmid DNA, using chemical transfection reagents, electroporation, or passive uptake depending on the cell type and application. The method of delivery greatly affects both on-target activity and off-target or non-specific toxicity. The best transfection approach relies on the cell type, particularly its ability to take up molecules, and the ASO chemistry, which affects cellular trafficking, stability, and nuclear targeting. The delivery method also determines the ASO dose; with active transfection, lower concentrations of 10–300 nM are often enough, whereas passive uptake usually needs doses in the micromolar range to reach similar intracellular levels and activity.^40^^,^^41^ The method of choice for ASO delivery into the cells does not seem to affect the subcellular distribution; both the transfection and the free uptake resulted in a similar localization pattern.^42^ However, Scharner et al.^43^ showed that delivering ASOs via free uptake reduced off-target activity compared with delivery via lipid transfection *in vitro*.^43^

Several methods were described for neuronal cell transfection with ASOs. Buijsen et al.^44^ compared various delivery methods, including lipid-based transfection, gymnotic uptake, Ca^2+^-enhanced medium (CEM)-based delivery, and electroporation, in 2D and 3D hiPSC-derived neuronal models.^44^ The authors showed that the CEM enhanced the delivery of splice-modulating ASOs, resulting in high transfection efficiency and low toxicity, rendering it suitable for ASO screening in neuronal cultures. The authors also noted a significant difference in cell-type-dependent transfection efficiency, with astrocytes being transfected at higher rates.

Shen et al.^45^ optimized the electroporation method for transfecting neuronal cultures by using a gapmer against the MALAT1 gene first and then applied the same protocol to induce the expression of the enzyme frataxin (FXN) in iPSC-derived human neuronal cells from patients with Friedreich’s ataxia.^45^ The method yielded an 80% knockdown efficiency for the MALAT1 gene, rescuing the FXN protein and enzyme activity with minimal cytotoxicity.

Lipid nanoparticles (LNPs) have been explored for ASO delivery in the CNS. Studies have shown that LNPs can encapsulate ASOs and facilitate their delivery to various CNS cell types *in vitro*. However, challenges remain regarding the optimization of LNP formulations to enhance ASO activity and distribution within the brain.^41^

For acute toxicity associated with ASO functionality and sequence specificity, doses in the nanomolar range were administered via lipid-based transfection methods. However, since lipid carriers have been shown to introduce additional toxicity by disrupting neuronal bioenergetics and mitophagy, which may confound the effects of the ASOs,^34^^,^^46^ the gymnotic delivery method was employed as an alternative in several studies. In this approach, doses ranging from 1 to 50 μM were used, replicating the *in vivo* IT administration, which results in a high and long-lasting ASO concentration in the cerebrospinal fluid (CSF) and brain.^14^

In addition to gymnosis, Umek et al.^47^ employed magnetofection to deliver ASOs into iPSC-derived neuronal stem cells from control and Huntington’s patients. The gene expression of the huntingtin gene was downregulated with both methods and did not compromise either the emergence of neural rosette structures or the self-renewal of neural stem cells (NSCs).^47^

While iPSC-derived neuronal cultures represent a central platform for evaluating target engagement and efficacy of CNS-directed ASOs, microglia warrant separate consideration. As the resident immune cells of the CNS, microglia exhibit distinct cellular biology, uptake mechanisms, and innate immune sensing pathways that critically influence ASO internalization, tolerability, and downstream responses. Unlike neurons, microglia are highly responsive to extracellular nucleic acids and experimental manipulations, including transfection and delivery strategies, which can induce activation or inflammatory phenotypes independent of target engagement. For instance, certain lipid or chemical transfection protocols induce cytokine release or stress responses in microglial cells, whereas other methods (e.g., magnetofection) mitigate such artifacts.^48^ This underscores the necessity of carefully selecting and validating delivery approaches when interpreting cytotoxicity and functional readouts in microglia. Vandermeulen et al.^49^ treated the hiPSC-derived microglia-like cells with ASOs directly in the culture medium. They observed rapid uptake of the oligonucleotides, reaching a plateau within about 60 min after addition.^49^

## Intracellular ASO detection methods for transfection efficiency

Several detection methods are available for ASOs. Still, each has its limitations, and there is a need for more comprehensive information on ASO uptake and distribution within cells and tissues. ASOs can be detected either directly or through the use of isotopic or fluorescent conjugates, enabling the detection of either the relative quantity of ASOs taken up by cells (northern blot and mass spectrometry) or providing more detailed information about their cellular distribution through the use of highly sensitive imaging methods (fluorescent labeling and fluorescence *in situ* hybridization [FISH]). Additionally, by monitoring substrate RNA or further downstream target protein expression using qPCR, western blot, or chemiluminescent assays, it is also possible to assess their efficiency and indirectly confirm their presence in cells.^50^

One of the earliest methods of ASO detection was radioactive labeling. The generation of such radioactive probes involves conjugation of iodine-125 (ˆ125I), indium-111 (^111^In), or phosphorus-32 (ˆ32 P) to the oligonucleotides.^51^^,^^52^ The use of ˆ125I-labeled ASOs has also been explored for their targeted therapeutic potential, especially in cancer treatment, by conjugating them with specific receptors such as vasoactive intestinal peptide (VIP), which facilitates selective targeting of tumor cells.^53^ The radioactive nature of these isotopes necessitates careful handling and disposal to ensure safety, which can be a limitation in clinical and large-scale research settings. The radiolabeling process can also introduce variability in ASO activity or stability, potentially affecting the interpretation of results.^54^

Non-radioactive isotope labeling has become an alternative approach to radiolabeling, where subcellular localization of^32^ S- or Br-labeled ASOs can also be achieved using nanoSIMS analysis.^55^ Fluorescent labeling of ASOs provides an easy detection method combined with highly sensitive microscopy techniques.^33^^,^^44^^,^^56^^,^^57^^,^^58^^,^^59^^,^^60^ Flow cytometry analysis (FACS) can also provide data on internalization by individual cells or cell types and quantitative measurement of their uptake.^61^ Chen et al.^33^ demonstrated the penetration efficacy of ASOs by labeling them with Cy5 and quantifying Cy5^+^ cells isolated from cortical organoids by FACS. Most cells, including CD90-expressing neurons, were Cy5^+^.^33^ On the other hand, labeling can result in the perturbation of oligomer properties, which could affect the binding efficiency of ASOs to their target RNA, leading to possible changes in the therapeutic efficacy. Additionally, fluorescent labels could introduce non-specific interactions with cellular components, complicating the interpretation of results related to the ASO’s activity and localization within the cells.^61^ More recently, proteins that are associated with the accumulation of PS-ASOs have been identified, providing a better understanding of the subcellular localization of PS-ASOs in combination with immunocytochemistry.^42^

*In situ* hybridization utilizes labeled probes for ASO detection, allowing for the direct visualization of ASO treatment within the cellular context without altering the properties of the target oligonucleotides. A disadvantage of this approach is that it can be technically challenging and time consuming, and effective hybridization and signal detection require precise conditions.^62^

A recently emerging version of this method is miRNAscope, which is a highly sensitive and robust *in situ* hybridization technique and can detect low-abundance miRNAs with spatial resolution. It allows for the detection of gene transcripts at the single-molecule level with single-cell resolution. However, specific probe generation can be costly, and the labeling process can be time consuming and labor intensive, making it less suitable for large-scale screenings.^40^^,^^63^

One possible avenue to overcome several limitations posed by the aforementioned detection methods is the use of antibodies specific for commonly used PS or 2′-MOE modifications, which, combined with immunofluorescence (IF) and confocal microscopy, have been shown to successfully detect the localization of gapmer ASOs in 3D neuronal spheroids and murine primary CNS cultures.^40^^,^^41^^,^^64^

The indirect detection methods for ASO uptake overlap with the approaches used to validate target modulation efficiency and potency. The two techniques most commonly employed across the studies are qPCR for RNA-level quantification^33^^,^^65^ and western blotting for protein-level validation.^44^

## Safety profiling of CNS ASOs using *in vitro* cytotoxicity assays

Beyond target engagement and efficacy, the early evaluation of safety is a critical component of CNS-targeted ASO development. *In vitro* cytotoxicity assays using neuronal cell cultures provide an essential first layer of safety profiling, enabling the detection of sequence-, chemistry-, and dose-dependent toxic effects before *in vivo* testing. An overview of *in vitro* cytotoxicity assays used to assess the safety of CNS ASOs in neuronal models is provided in Tables 3 and 4. Hagedorn et al.^68^ evaluated the acute neurotoxic potential of 1,825 gapmer ASOs with LNA-modified flanks and a central DNA region supporting RNase H binding.^68^ The authors monitored the impact of ASOs on spontaneous calcium oscillations of rat primary cortical neuronal cultures and found that it accurately predicted the acute neurotoxic potential in mice. They have found that the sequence features responsible for the toxic potential of the ASOs are the number and position of guanine (G) nucleotides relative to the 3′ end of the ASO, which increases the toxic potential of the ASO, and the number of adenine nucleotides, which decreases it.

**Table 3:** *In vitro* cytotoxicity assays employed for the safety assessment of CNS ASOs in neuronal cultures

Toxicity	Method	Cell model	ASO type	Controls	Dose	Output	Biological interpretation
General cytotoxicity	lactate dehydrogenase (LDH) release assay^12^^,^^66^^,^^67^	patient iPSC-derived neurons	LNA, PS gapmers	control iPSC-derived neurons, scrambled ASO	5 μM	extracellular LDH	membrane integrity
		Neuro-2a, BE(2)-M17	2′-OMe and 5′-CP gapmers	LNA, PS gapmers	50 nM		
		primary murine microglia	2′-MOE, PS gapmers	scrambled ASOs	30–50 nM		
	ATP viability assay^34^	patient iPSC-derived neurons	2′-MOE, PS ssASOs	control iPSC-derived neurons, untreated, scrambled ASO	1–300 nM	cellular ATP	metabolic energy
	live-cell imaging^44^	control iPSC-derived neurons and organoids	2′-MOE, PS ssASOs	untreated	10–500 nM	cell count	cell survival
		Neuro-2a, BE(2)-M17	2′-OMe and 5′-CP gapmers	LNA, PS gapmers	50 nM		
	alamarBlue^50^	primary murine neurons	LNA, PS gapmers	scrambled ASO	0.02–1.25 μM	resazurin reduction	mitochondrial respiration
Apoptosis	c-Cas3 imaging with immunocytochemistry^33^	patient-derived cortical organoids	2′-MOE, PS ssASOs	control-derived cortical organoid, scrambled ASO	10 μM	c-Cas3 visualization	c-Cas3 nuclear translocation indicates apoptosis

**Table 4:** Neurotoxicity assays employed for the safety assessment of CNS ASOs in neuronal cultures

Toxicity	Method	Cell model	ASO type	Controls	Dose	Output	Biological interpretation
Neurotoxicity	spontaneous calcium oscillations^68^	rat primary neurons	LNA-modified PS ASO	untreated, non-toxic ASO	25 μM	pattern of calcium oscillations	altered calcium oscillations predict functional network neurotoxicity
	intracellular free calcium^69^	rat primary neurons	gapmers with PS backbone	untreated, non-toxic ASO	3.1–25 μM	concentration of intracellular calcium	elevation predicts excitotoxicity

Furthermore, Jia et al.^69^ demonstrated that toxic ASOs described previously^68^ significantly decreased intracellular free calcium levels in rat primary neuronal cultures, while *in vivo*, these toxic ASOs were found to reduce consciousness and locomotor function in mice in a dose-dependent manner, confirming that changes in intracellular free calcium levels are a part of the mechanism underlying the neurotoxic effects of ASOs. During *in vivo* administration of ASOs preformulated with Ca^2+^ ions, they observed a slight increase (∼10%–15%) in observable short-term cumulative neurotoxicity measured by electrophysiology.^69^

Contrary to that, subsequent studies by Miller et al.^70^ demonstrated the opposite, with a decreased acute neurotoxicity of CNS-administered ASOs with the use of aCSF supplemented with Ca^2+^ and Mg^2+^. This could be explained by them using a higher dosage, 225 μg, in comparison with the 25 μg from the aforementioned study, thus demonstrating more severe toxic effects after ASO treatment, such as seizures and death rather than effects on consciousness, motor function, and appearance. Additionally, they confirmed higher toxicity in gapmer ASOs with a full PS backbone and found mixed PO/PS backbones to induce lower toxic effects. Nonetheless, the supplemented aCSF reduced toxicity with both formulations. Extended studies are required to fully understand the differences between the studies.^70^ Furthermore, O'Rourke et al.^11^ tested the acute neuronal inhibition response caused by PS gapmer ASOs following local delivery to the CNS in C57BL/6 mice and Sprague-Dawley rats. They confirmed that acute inhibition is associated with neuronal transmission disruption. It is transient, reversible, and dose dependent after CNS ASO administration and translates across species. Sequences rich in guanine with low cytosine content showed more severe acute inhibition, while reduction in PS backbone modifications mitigated it, and 2′ sugar modifications did not influence acute inhibition. *In vitro* (multielectrode array (MEA) assays in mouse primary cortical cultures found that the same ASOs causing acute inhibition *in vivo* suppressed spontaneous field potentials *in vitro*. Another comparative experiment confirmed that removing ASOs from culture media restored spontaneous excitation in neurons. They found that excitatory stimuli *in vitro*, e.g., glutamate or acetylcholine, can reverse ASO-mediated inhibition, which indicates that non-specific binding of the ASOs to receptors could be the cause of acute inhibition.^11^ These studies confirm the potential of sequence, chemistry, and formulation modifications in mitigating neurotoxicity of ASOs, with the PS backbone modification being highly important. These *in vivo* and *in vitro* rodent studies demonstrate toxic effects relevant to humans, as the CNS of rodents mimics closely the human CNS. Further studies in hiPSC-derived models could solidify these data and aid in the understanding of these parameters in further ASO development.

Chen et al.^33^ described the design and screening of ASOs aimed at promoting the inclusion of exon 8 in favor of exon 8A in the CACNA1C isoforms in human neural cells as a potential treatment for Timothy syndrome. To assess the cytotoxicity of these ASOs, the authors employed the TUNEL assay and cleaved caspase-3 (c-Cas3) staining to detect apoptosis in neurons derived from cortical organoids matured up to day 210. The cytotoxicity tests were conducted on monolayers obtained after the dissociation and culturing of the organoids using 10 μM ASO and passive uptake for 2 days.^33^

Several studies assess ASO-induced toxicity using the lactate dehydrogenase (LDH) release assay in combination with morphological characterization. The LDH-release assay measured from the culture medium detects loss of plasma membrane integrity. It is therefore a direct indicator of cell death, particularly necrosis or late-stage apoptosis. Hauser et al.^66^ evaluated potential toxic effects of ASO treatment on SCA3 iPSC-derived neurons by measuring LDH activity in the culture supernatant at 14, 21, and 28 days post-treatment. Consistent with these findings, immunocytochemical TUJ staining of SCA3 neurons revealed no evident morphological alterations following ASO treatment, with a stable and robust neuronal network maintained.^66^

A study published by Kuroda et al.^12^ showed that strategic chemical modifications of ASOs markedly reduced LDH release compared with unmodified counterparts, highlighting the contribution of ASO chemistry to cytotoxicity.^12^ Furthermore, the authors also described that neurotoxic ASOs induce the mislocalization of paraspeckle proteins in neuronal cells and cause the upregulation of the p53-regulated transcripts to trigger cell death. The LDH release assay was also used by Braatz et al.^67^ to analyze the effects of ASOs against NLRP3 on the viability of primary murine microglia.^67^ In contrast to the LDH-release assay, alamarBlue measures cellular metabolic activity, based on the reduction of resazurin to resorufin by viable cells. It reflects cell viability and metabolic competence, rather than cell death per se. It was used by Didiot et al.^50^ to measure the cytotoxicity of various ASO concentrations against Huntingtin mRNA in primary cortical neurons.^50^

## Off-target effects

ASOs can have off-target effects that can either arise from their chemical properties (class effects) or their specific sequences.^5^ These sequence-specific effects are categorized into effects that are independent of the ASO binding to the RNA (hybridization-independent effects) and those dependent on binding to the RNA (hybridization-dependent effects). The evaluation of potential off-target effects—using both computational prediction tools and experimental validation—has become a key component of the ASO development pipeline.^19^^,^^43^^,^^71^ The most relevant approaches for off-target detection are listed in Table 5. Hybridization-dependent off-target effects occur when ASOs bind to unintended RNA sequences that share homology with the target sequence.^43^^,^^71^^,^^74^ These effects can theoretically be predicted through *in silico* sequence homology assessments but are often underestimated due to limitations in commonly used prediction tools. For example, the BLAST algorithm, which is frequently applied to identify potential ASO sequence complementarity, heavily penalizes gaps and bulges and does not account for G:U wobble base pairing, a frequent feature of RNA secondary structures.^72^ As a result, biologically relevant partial or imperfect ASO-RNA interactions may be overlooked, leading to an underestimation of potential off-target binding events. Recent machine learning-assisted platforms have begun to streamline ASO design by improving sequence selection and predicting functional outcomes. According to FDA guidelines, *in silico* models aimed to address efficacy and specificity by improving ASO design present a valuable tool for prediction of off-target binding.^28^ For example, eSkipFinder uses machine learning models trained on experimental data to prioritize exon-skipping ASO candidates,^75^ and ASOptimizer applies deep learning to score and optimize ASO sequences along with chemical modification patterns, enhancing predicted efficacy for specific RNA targets such as *IDO1* (e.g., for silencing studies).^73^ Other computational pipelines like ASOG automate ASO generation and evaluation by integrating structure prediction and hybridization parameters.^76^ As CNS-acting ASOs cannot permeate the BBB and are usually injected intrathecally, there is a need to develop *in silico* tools able to analyze uptake and delivery properties. A recent study by Linninger et al.^77^ introduced a distributed mechanistic pharmacokinetics (DMPK) model in non-human primates to serve as a computational platform to simulate IT administration and enable predictive analysis of drug pharmacokinetics in the CNS, modeling ASO interactions with neurons and receptors and tracking ASO biodistribution.^77^

**Table 5:** ASO off-target effect detection methods with CNS relevance

Method	Type of off-target detected	Sensitivity	Specificity	Throughput
*In silico* (BLAST/MFE)	hybridization dependent^72^^,^^73^	medium (misses partial hybrids)	low (high false positives)	very high (genome-wide instant)
Immune/cytokine assays (ELISA, PBMC)	immunostimulation (TLR activation)^33^^,^^34^	high	high	high
RNA-seq	hybridization dependent and independent^65^	high	medium	medium
Targeted RT-qPCR	prioritized candidates^33^	high for known target	high	low
Protein binding	hybridization independent^42^	high	medium	low

Following *in silico* evaluation, RNA sequencing (RNA-seq) is adopted as the standard method to confirm predicted off-target hybridization of ASOs, which is also endorsed by the newest FDA guidance.^28^ Direct and secondary off-target effects can be differentiated through a time-course RNA-seq or by using a cell line with a knockout of the target gene. There is overall a shortage of publicly available, standardized, and high-quality RNA-seq datasets, since private pharmaceutical companies keep their information, and there is high variability in terms of cell lines, delivery methods, and chemical modifications of the ASOs.

Complementary to RNA-seq, proteomic analysis can reveal hybridization-independent off-target effects. These include immune activation, thrombocytopenia,^78^ complement activation, and coagulation inhibition, which are induced by sequence motifs such as CpG or the PS modification binding to intracellular and extracellular proteins. After ASO administration *in vitro*, the ASO-protein complexes are isolated from the cells and identified with mass spectrometry, followed by proteomic analysis. Proteins following patterns of RNA expression indicate hybridization-dependent binding, while protein expression levels contradicting the RNA expression levels are caused by hybridization-independent binding. Since protein half-lives are varied, a time-course analysis helps differentiate acute binding from transcript-driven changes.^79^

Considering hybridization-independent effects activate TLR9, there are models based on assessing TLR9 activation upon ASO treatment.^80^ At the CNS level, the main cell types expressing the TLR9 receptor are microglia and astrocytes. Despite the availability of both cell types through iPSC technology, there are currently no published studies assessing immune activation induced by CNS-targeting ASOs in these cells. Nevertheless, there are studies on their immune activation in mono and co-cultures such as the one by Tujula et al.^36^ To date, predictive immunogenicity testing has relied primarily on TLR9 reporter HEK293 cells to measure NF-κB-dependent TLR9 signaling^33^ or on human peripheral blood mononuclear cell (PBMC) assays, in which cytokine secretion is quantified following ASO treatment.^34^

hiPSC-derived microglia and astrocytes display high variability and heterogeneity, which can reflect in the innate expression levels of TLR9. One of the main hurdles is having mature cell lines, as monocultures can retain a fetal transcriptomic profile. They require a complex protocol of culturing in comparison with PBMCs, making them also more complicated for high-throughput screening in comparison with HEK293 cell lines. However, their biological relevance gives them an important role in future *in vitro* screening developments. To overcome their setbacks, co-cultures, cerebral organoids, or organ-on-a-chip culturing could create an environment similar to the human brain. During differentiation, the addition of specific differentiation factors like IL-34, CSF-1, and TGF-β1 to microglia is crucial. The use of commercially available astrocyte induction medium (AIM) and astrocyte maturation medium (AMM) complemented with ciliary neurotrophic factor (CNTF) would ensure proper differentiation of astrocytes. In order to validate hiPSC-derived microglial models, functional tests like phagocytosis and expression of markers like TMEM119, P2RY12, CX3CR1, and IBA1 are necessary. For hiPSC-derived astrocytes, Ca^2+^ imaging and expression of GFAP, S100B, ALDH1L1, and GLT-1 are significant to confirm successful differentiation.^36^^,^^81^ Tables 6^12^^,^^32^^,^^33^^,^^34^^,^^35^^,^^36^^,^^37^^,^^38^^,^^39^^,^^50^^,^^68^^,^^66^^,^^67^^,^^69^, 7,^32^^,^^33^^,^^34^^,^^35^^,^^36^^,^^37^^,^^38^^,^^39^^,^^42^^,^^65^^,^^72^^,^^73^, and 8,^82^present general guidelines for analyzing aspects of common toxicity assays for CNS ASOs. A key aspect in general *in vitro* toxicity assays for CNS ASOs is the correct standardized controls, which must include an untreated control, vehicle/transfection reagent control, scrambled ASO control, non-targeting ASO with identical chemistry, multiple biological replicates (≥3), and control cell lines. Apart from those, recommended controls are positive cytotoxic controls and/or positive inflammatory controls, which are highly dependent on each assay. Overall, the key steps to setting up an *in vitro* toxicity platform for ASOs are the following:

(1) Set up an *in vitro* platform by establishing cell lines, controls, and testing ASO integrity.
(2) Start with general toxicity assays such as viability assays and continue with cell-type-specific assays like measuring spontaneous calcium oscillations in neurons and immunotoxicity assays in microglia and astrocytes.
(3) Determine the dose response and IC_50_ for assays.
(4) Validate results across multiple biological repeats and multiple platforms.
(5) Continue with more complex and physiologically relevant models, such as highly matured iPSC-derived neurons and co-cultures.
(6) Once the assays are validated and established properly, testing the most complex models in the form of organoids and assembloids can be used to test chronic toxicity and distribution of ASOs in the systems in detail.
(7) Consistently reporting dose ranges, controls, and IC_50_ methodology leads to overall improved reproducibility.

**Table 6:** General guidelines about common assays utilized for testing *in vitro* toxicity of CNS ASOs: neurotoxicity, cytotoxicity, apoptosis, and astrogliosis

	Experiment	Assays	Controls	Cost	Availability	Practical considerations
Preliminary experiments	ASO validation	(RP-) HPLC, stability, solubility	vehicle only	moderate	moderate to high	critical to determine ASO integrity
	standardized viability	MTT, alamarBlue, ATP, LDH	standardized controls, positive cytotoxic control	low to moderate	high	high concentration range, ≥3 biological repeats
	IC_50_ calculation	nonlinear regression based on viability assay	standardized controls, positive cytotoxic control	low	high	ensure appropriate dose range to avoid extrapolated values, multiple time points
Neurotoxicity	excitotoxicity and network disruptions	intracellular free calcium	standardized controls, stimulant/inhibitor	moderate	moderate to high	measures live kinetics and acute neurotoxicity; requires advanced plate readers/platforms
		spontaneous calcium oscillations		moderate	moderate to high	
		microelectrode array		high	moderate	
Cytotoxicity	metabolic energy	ATP (adenosine triphosphate) assay	standardized controls, positive cytotoxic control	moderate	high	time sensitive due to ATP degradation; optimal usually 24 after ASO treatment
	membrane integrity	LDH (lactate dehydrogenase) release assay	standardized controls, positive cytotoxic control	low	high	time sensitive due to LDH secretion; optimal usually 72 h after ASO treatment
Apoptosis	cell death mechanism	caspase assays, TUNEL, Annexin V/PI	standardized controls, positive apoptotic control	moderate	high	time sensitive (caspase, 24 h; TUNEL, 72 h after ASO treatment)
Astrogliosis	GFAP and vimentin quantification	ELISA, immunocytochemistry	standardized controls, positive reactivity control	low to moderate	high	maturation and heterogeneity of astrocytes, spontaneous activation

**Table 7:** General guidelines about common assays utilized for testing *in vitro* toxicity of CNS ASOs: immunotoxicity, transcriptomics, and proteomics

Immunotoxicity	inflammation	ELISA, cytokine array	standardized controls, proinflammatory control	moderate	high	time sensitive; optimal between 2 and 24 h after ASO treatment as cytokines are degraded or they accumulate extensively, overly sensitive microglia or astrocyte monocultures
	phagocytosis	live-cell phagocytosis assay	standardized controls, inhibitor	moderate	moderate to high	measures live cells; requires system to keep cells in physiological conditions during the time course
Transcriptomics	full transcriptome sequencing	RNA-seq	standardized controls	moderate	moderate	requires specialized equipment and bioinformatic skills, RNA instability
	specific target gene analysis	qPCR	standardized controls	high	moderate	requires specialized equipment, RNA instability
Proteomics	ASO-protein hybridization	LC-MS, proteomic analysis	standardized controls	high	low to moderate	requires specialized equipment and bioinformatic skills, extraction and treatment of proteins is highly sensitive

**Table 8:** Dose-range recommendations for IC_50_ determination

Number of concentrations	≥8
Spacing	2-fold or half-logarithmic serial dilutions
Dose range	wide enough to produce 0%–100% response (or maximal measurable effect)
Replicates	≥3 biological replicates
Exposure	one or multiple when needed
Confidence interval	95%
Curve	four-parameter logistic regression

## Future directions in CNS-targeted ASO therapeutics

ASO therapeutics have progressed in the past decade, and two have transitioned from experimental modalities to clinically validated interventions for CNS disorders. While early outcomes have established target engagement and disease-modifying potential, future development in the field depends on changes in delivery, molecular precision, and durability of therapeutic effect. Market participants such as Ionis Pharmaceuticals and Biogen have demonstrated the clinical application of intrathecally delivered therapies for SMA (nusinersen, Spinraza) and SOD1-associated ALS (tofersen, Qalsody).

Alongside these developments, multiple ASOs have failed during development or demonstrated adverse effects post-administration. One such example is valeriasen designed for the treatment of severe KCNT1 epileptic encephalopathy. Despite promising results during *in silico*, *in vitro*, and *in vivo* safety and toxicity assessments, the treatment was associated with the development of hydrocephalus in patient 1. Following this adverse effect, the patient was deceased 3 months after their final ASO dose. Patient 2 received the same ASO under closer clinical monitoring, allowing for the management of the hydrocephalus. This patient currently experiences a 66% reduction in seizures.^83^ Examples like these indicate a requirement for deeper mechanistic investigation, underscoring the role of screening, risk assessment, and proactive clinical monitoring and management.

The next generation of CNS ASOs is focused on allele-selective and mutation-specific strategies, particularly in dominantly inherited neurodegenerative diseases such as Huntington’s disease and select forms of ALS and Parkinson’s disease.^84^^,^^85^^,^^86^ Requirements that need to be evaluated during the development of these therapies are stereopure chemistries and backbone modifications to alter potency, safety, and target specificity. An important area of focus lies in addressing the limitations of CNS delivery and dosing frequency. Emerging BBB shuttle technologies, including receptor-mediated transport systems under development by companies such as Roche/Genentech and Denali Therapeutics, aim to enable systemic administration and broader brain distribution. In parallel, long-acting delivery platforms, implantable depots, and chemically modified ASOs are being explored to extend dosing intervals and modify patient adherence, particularly in chronic neurodegenerative conditions.

Beyond monogenic disorders, CNS ASO development is expanding toward complex and multifactorial diseases, including Alzheimer’s disease, Parkinson’s disease, and neuroinflammatory conditions. This shift requires biomarker integration, earlier intervention strategies, and patient stratification to manage therapeutic impact. The field is moving toward a model in which CNS ASOs are target specific, delivery optimized, durable, and scalable.

## Conclusion

ASO therapeutics have emerged as a powerful and versatile modality for the treatment of neurological disorders, driven by advances in chemical modification, delivery strategies, and a growing understanding of their pharmacokinetics and pharmacodynamics in the CNS. Clinical successes such as nusinersen and tofersen have validated intrathecally delivered ASOs as effective interventions for CNS diseases and have further enabled the development of highly individualized, n-of-1 therapeutic approaches for ultra-rare genetic conditions.

Despite this progress, the development of CNS-directed ASOs remains constrained by challenges related to delivery, safety, and off-target effects. While chemical modifications enhance stability and potency, they also influence protein binding, immune activation, and sequence-dependent neurotoxicity, underscoring the need for early and comprehensive safety profiling. *In vitro* neuronal and glial models—particularly those derived from patient-specific iPSCs—together with organoid and assembloid systems, have become indispensable tools for evaluating target engagement, cytotoxicity, and sequence-dependent liabilities before *in vivo* testing. Importantly, accumulating evidence demonstrates that sequence features, chemistry, and formulation can predict acute neurotoxicity and neuroexcitability with reasonable accuracy, highlighting the value of systematic *in vitro* screening to deselect high-risk ASOs early in development.

However, the recent experience with a KCNT1-targeted ASO, in which delayed CNS toxicity emerged despite reassuring *in silico*, *in vitro*, and *in vivo* safety data, illustrates that current platforms do not yet reliably predict all clinically relevant outcomes. This gap is compounded by the fact that, many current *in vitro* studies rely on limited dose ranges and lack standardized positive and negative controls, complicating the interpretation of toxicity and potency data and often precluding the determination of key pharmacological parameters such as IC_50_ values. The incorporation of broader dose-response analyses, appropriate scrambled or mock controls, and reference toxic ASOs will be critical to improving comparability across studies and strengthening translational relevance. Moreover, given the distinct biology and innate immune sensing properties of different CNS cell types, particularly microglia and astrocytes, safety and efficacy assessments should increasingly be performed in cell-type-specific and multicellular systems rather than relying solely on neuronal monocultures.

Closing this gap will also require closer alignment between preclinical safety packages and the evolving regulatory landscape, including ICH S13 and recent FDA and EMA guidance on oligonucleotide-specific risks. Looking ahead, the integration of advanced *in vitro* platforms, including 3D cultures, brain organoids, assembloids, and co-culture systems, together with improved computational prediction and proteomic off-target detection tools, holds promise for refining ASO design and safety assessment. Such approaches are especially crucial for personalized and n-of-1 ASO therapies, where traditional preclinical pipelines are not feasible. Ultimately, rigorous *in vitro* screening in disease- and cell-type-relevant models, informed by both mechanistic understanding and hard-won clinical lessons, will be essential to maximize therapeutic benefit, minimize risk, and support the continued expansion of ASO-based treatments for CNS disorders.

## Acknowledgments

This project is funded by the EU’s 10.13039/100018693Horizon Europe MSCA scheme, grant agreement no. 101120256 (MMM).

## Author contributions

L.C.Z., R.A.-S., and A.B. contributed to manuscript preparation. A.B. conceptualized the study and led the writing. W.M.C.v.R.-M. and A.D. supervised the project and secured funding.

## Declaration of interests

The authors declare no competing interests.

## References

[1] Lauffer M.C., van Roon-Mom W., Aartsma-Rus A., N = 1 Collaborative (2024). Possibilities and limitations of antisense oligonucleotide therapies for the treatment of monogenic disorders. Commun. Med..

[2] Aartsma-Rus A. (2017). FDA Approval of Nusinersen for Spinal Muscular Atrophy Makes 2016 the Year of Splice Modulating Oligonucleotides. Nucleic Acid Ther..

[3] Blair H.A. (2023). Tofersen: First Approval. Drugs.

[4] Kim J., Hu C., Moufawad El Achkar C., Black L.E., Douville J., Larson A., Pendergast M.K., Goldkind S.F., Lee E.A., Kuniholm A. (2019). Patient-Customized Oligonucleotide Therapy for a Rare Genetic Disease. N. Engl. J. Med..

[5] Kim J., Woo S., de Gusmao C.M., Zhao B., Chin D.H., DiDonato R.L., Nguyen M.A., Nakayama T., Hu C.A., Soucy A. (2023). A framework for individualized splice-switching oligonucleotide therapy. Nature.

[6] Belgrad J., McConnell E., Leonard S., Nolen N., Lauffer M.C., Watts J.K., Yu T., Yan W.X., Aartsma-Rus A. (2025). The N=1 Collaborative: advancing customized nucleic acid therapies through collaboration and data sharing. Nucleic Acids Res..

[7] Stephenson M.L., Zamecnik P.C. (1978). Inhibition of Rous sarcoma viral RNA translation by a specific oligodeoxyribonucleotide. Proc. Natl. Acad. Sci. USA.

[8] Khvorova A., Watts J.K. (2017). The chemical evolution of oligonucleotide therapies of clinical utility. Nat. Biotechnol..

[9] Quemener A.M., Centomo M.L., Sax S.L., Panella R. (2022). Small Drugs, Huge Impact: The Extraordinary Impact of Antisense Oligonucleotides in Research and Drug Development. Molecules.

[10] De Serres-Bérard T., Ait Benichou S., Jauvin D., Boutjdir M., Puymirat J., Chahine M. (2022). Recent Progress and Challenges in the Development of Antisense Therapies for Myotonic Dystrophy Type 1. Int. J. Mol. Sci..

[11] O'Rourke J.G., Bachmann G., Mazur C., Zhou K., Platoshyn O., Bravo Hernandez M., Klein S.K., Nguyen J., Burel S., Hoffmaster C. (2026). Acute neuronal inhibition response caused by phosphorothioate antisense oligonucleotides following local delivery to the central nervous system. Nucleic Acids Res..

[12] Kuroda T., Yoshioka K., Mon S.S.L., Katsuyama M., Sato K., Isogai E., Yoshida-Tanaka K., Iwata-Hara R., Yamaguchi T., Obika S., Yokota T. (2025). Unraveling and controlling late-onset neurotoxicity of antisense oligonucleotides through strategic chemical modifications. Mol. Ther. Nucleic Acids.

[13] Ling K., Prakash T.P., Yu J., Jackson M., Chun S.J., Bachmann G., Post N., Greenlee S., Soriano A., Hunyara J.L. (2026). Enhanced splicing modulation by NMA-modified antisense oligonucleotides. Nucleic Acids Res..

[14] Mazur C., Powers B., Zasadny K., Sullivan J.M., Dimant H., Kamme F., Hesterman J., Matson J., Oestergaard M., Seaman M. (2019). Brain pharmacology of intrathecal antisense oligonucleotides revealed through multimodal imaging. JCI Insight.

[15] Jafar-Nejad P., Powers B., Soriano A., Zhao H., Norris D.A., Matson J., DeBrosse-Serra B., Watson J., Narayanan P., Chun S.J. (2021). The atlas of RNase H antisense oligonucleotide distribution and activity in the CNS of rodents and non-human primates following central administration. Nucleic Acids Res..

[16] Rigo F., Chun S.J., Norris D.A., Hung G., Lee S., Matson J., Fey R.A., Gaus H., Hua Y., Grundy J.S. (2014). Pharmacology of a central nervous system delivered 2'-O-methoxyethyl-modified survival of motor neuron splicing oligonucleotide in mice and nonhuman primates. J. Pharmacol. Exp. Ther..

[17] Finkel R.S., Chiriboga C.A., Vajsar J., Day J.W., Montes J., De Vivo D.C., Yamashita M., Rigo F., Hung G., Schneider E. (2016). Treatment of infantile-onset spinal muscular atrophy with nusinersen: a phase 2, open-label, dose-escalation study. Lancet.

[18] Andersson S., Antonsson M., Elebring M., Jansson-Löfmark R., Weidolf L. (2018). Drug metabolism and pharmacokinetic strategies for oligonucleotide- and mRNA-based drug development. Drug Discov. Today.

[19] Gagnon K.T., Corey D.R. (2019). Guidelines for Experiments Using Antisense Oligonucleotides and Double-Stranded RNAs. Nucleic Acid Ther..

[20] Stoker T.B., Andresen K.E.R., Barker R.A. (2021). Hydrocephalus Complicating Intrathecal Antisense Oligonucleotide Therapy for Huntington's Disease. Mov. Disord..

[21] Machetanz G., Grziwotz M., Semmler L., Maier M., Maegerlein C., Deschauer M. (2023). Symptomatic intracranial hypertension in an adult patient with spinal muscular atrophy and arachnoid cysts receiving nusinersen. J. Neuromuscul. Dis..

[22] Kilanowska A., Studzińska S. (2020). In vivo and in vitro studies of antisense oligonucleotides - a review. RSC Adv..

[23] Jonker A.H., Tataru E.A., Graessner H., Dimmock D., Jaffe A., Baynam G., Davies J., Mitkus S., Iliach O., Horgan R. (2025). The state-of-the-art of N-of-1 therapies and the IRDiRC N-of-1 development roadmap. Nat. Rev. Drug Discov..

[24] Aartsma-Rus A., van Roon-Mom W., Lauffer M., Siezen C., Duijndam B., Coenen-de Roo T., Schüle R., Synofzik M., Graessner H. (2023). Development of tailored splice-switching oligonucleotides for progressive brain disorders in Europe: development, regulation, and implementation considerations. RNA.

[25] Schüle R., Graessner H., Aartsma-Rus A., van Roon-Mom W.M.C., N=1 Collaborative N1C, 1 Mutation 1 Medicine Consortium 1M1M, Synofzik M. (2025). Tailored antisense oligonucleotides for ultrarare CNS diseases: An experience-based best practice framework for individual patient evaluation. Mol. Ther. Nucleic Acids.

[26] International Council for Harmonisation (2009). Guideline on nonclinical safety studies for the conduct of human clinical trials. https://database.ich.org/sites/default/files/M3_R2__Guideline.pdf.

[27] International Council for Harmonisation (2024). Nonclinical Safety Assessment of Oligonucleotide-Based Therapeutics Guidance for Industry. https://database.ich.org/sites/default/files/ICH_S13EWG_Concept_Paper_2024_1028.pdf.

[28] U.S. Food and Drug Administration (2024). Final Concept Paper S13 EWG: Non-clinical Safety Evaluation of Oligonucleotide-based Therapeutics. https://www.fda.gov/media/183496/download.

[29] U.S. Food and Drug Administration (2021). Nonclinical Testing of Individualized Antisense Oligonucleotide Drug Products for Severely Debilitating or Life-Threatening Diseases Guidance for Sponsor-Investigators. https://www.fda.gov/media/147876/download.

[30] European Medicines Agency (2024). Guideline on the Development and Manufacture of Oligonucleotides (EMA/CHMP/CVMP/QWP/262313/202424). https://www.ema.europa.eu/en/documents/scientific-guideline/draft-guideline-development-manufacture-oligonucleotides_en.pdf.

[31] European Medicines Agency (2026). Non-clinical guidelines: toxicology. https://www.ema.europa.eu/en/human-regulatory-overview/research-development/scientific-guidelines/non-clinical-guidelines/non-clinical-guidelines-toxicology.

[32] Zuccaro M.V., Young R.E., Hu J., Lanzano P., Semenova E., Lin X., Skowronski A., Shen Y., Kim T.H., Miller D.E. (2026). Antisense oligonucleotides to KIF1A polymorphisms expand targets and rescue patient-derived neurons in vitro. Nat. Commun..

[33] Chen X., Birey F., Li M.Y., Revah O., Levy R., Thete M.V., Reis N., Kaganovsky K., Onesto M., Sakai N. (2024). Antisense oligonucleotide therapeutic approach for Timothy syndrome. Nature.

[34] Williams L.A., Gerber D.J., Elder A., Tseng W.C., Baru V., Delaney-Busch N., Ambrosi C., Mahimkar G., Joshi V., Shah H. (2022). Developing antisense oligonucleotides for a TECPR2 mutation-induced, ultra-rare neurological disorder using patient-derived cellular models. Mol. Ther. Nucleic Acids.

[35] Ziegler A., Carroll J., Bain J.M., Sands T.T., Fee R.J., Uher D., Kanner C.H., Montes J., Glass S., Douville J. (2024). Antisense oligonucleotide therapy in an individual with KIF1A-associated neurological disorder. Nat. Med..

[36] Tujula I., Hyvärinen T., Lotila J., Rogal J., Voulgaris D., Sukki L., Tornberg K., Korpela K., Jäntti H., Malm T. (2025). Modeling neuroinflammatory interactions between microglia and astrocytes in a human iPSC-based coculture platform. Cell Commun. Signal..

[37] Shayestehfar M., Taherkhani T., Jahandideh P., Hamidieh A.A., Faramarzpour M., Memari A. (2025). Brain tumors and induced pluripotent stem cell technology: a systematic review of the literature. Ann. Med. Surg..

[38] Lange J., Zhou H., McTague A. (2022). Cerebral Organoids and Antisense Oligonucleotide Therapeutics: Challenges and Opportunities. Front. Mol. Neurosci..

[39] Means J.C., Martinez-Bengochea A.L., Louiselle D.A., Nemechek J.M., Perry J.M., Farrow E.G., Pastinen T., Younger S.T. (2025). Rapid and scalable personalized ASO screening in patient-derived organoids. Nature.

[40] Fial I., Farrier S.A., Chimento D.P., Ascoli C.A., Wan X., Oliver P.L. (2025). Characterizing Antibodies Targeting Antisense Oligonucleotide Phosphorothioate and 2'-O-Methoxyethyl Modifications for Intracellular Trafficking and Biodistribution Studies. Nucleic Acid Ther..

[41] Byrnes A.E., Dominguez S.L., Yen C.W., Laufer B.I., Foreman O., Reichelt M., Lin H., Sagolla M., Hötzel K., Ngu H. (2023). Lipid nanoparticle delivery limits antisense oligonucleotide activity and cellular distribution in the brain after intracerebroventricular injection. Mol. Ther. Nucleic Acids.

[42] Crooke S.T., Wang S., Vickers T.A., Shen W., Liang X.H. (2017). Cellular uptake and trafficking of antisense oligonucleotides. Nat. Biotechnol..

[43] Scharner J., Ma W.K., Zhang Q., Lin K.T., Rigo F., Bennett C.F., Krainer A.R. (2020). Hybridization-mediated off-target effects of splice-switching antisense oligonucleotides. Nucleic Acids Res..

[44] Buijsen R.A.M., van der Graaf L.M., Kuijper E.C., Pepers B.A., Daoutsali E., Weel L., Raz V., Parfitt D.A., van Roon-Mom W.M.C. (2024). Calcium-Enhanced Medium-Based Delivery of Splice Modulating Antisense Oligonucleotides in 2D and 3D hiPSC-Derived Neuronal Models. Biomedicines.

[45] Shen X., Beasley S., Putman J.N., Li Y., Prakash T.P., Rigo F., Napierala M., Corey D.R. (2019). Efficient electroporation of neuronal cells using synthetic oligonucleotides: identifying duplex RNA and antisense oligonucleotide activators of human frataxin expression. RNA.

[46] Napoli E., Liu S., Marsilio I., Zarbalis K., Giulivi C. (2017). Lipid-based DNA/siRNA transfection agents disrupt neuronal bioenergetics and mitophagy. Biochem. J..

[47] Umek T., Olsson T., Gissberg O., Saher O., Zaghloul E.M., Lundin K.E., Wengel J., Hanse E., Zetterberg H., Vizlin-Hodzic D. (2021). Oligonucleotides Targeting DNA Repeats Downregulate Huntingtin Gene Expression in Huntington's Patient-Derived Neural Model System. Nucleic Acid Ther..

[48] Carrillo-Jimenez A., Puigdellívol M., Vilalta A., Venero J.L., Brown G.C., StGeorge-Hyslop P., Burguillos M.A. (2018). Effective Knockdown of Gene Expression in Primary Microglia With siRNA and Magnetic Nanoparticles Without Cell Death or Inflammation. Front. Cell. Neurosci..

[49] Vandermeulen L., Geric I., Fumagalli L., Kreir M., Lu A., Nonneman A., Premereur J., Wolfs L., Policarpo R., Fattorelli N. (2024). Regulation of human microglial gene expression and function via RNAase-H active antisense oligonucleotides in vivo in Alzheimer's disease. Mol. Neurodegener..

[50] Didiot M.C., Ferguson C.M., Ly S., Coles A.H., Smith A.O., Bicknell A.A., Hall L.M., Sapp E., Echeverria D., Pai A.A. (2018). Nuclear Localization of Huntingtin mRNA Is Specific to Cells of Neuronal Origin. Cell Rep..

[51] Dewanjee M.K., Ghafouripour A.K., Werner R.K., Serafini A.N., Sfakianakis G.N. (1991). Development of sensitive radioiodinated anti-sense oligonucleotide probes by conjugation technique. Bioconjug. Chem..

[52] Metz T., Welling M.M., Suidgeest E., Nieuwenhuize E., de Vlaam T., Curtis D., Hailu T.T., van der Weerd L., van Roon-Mom W.M.C. (2024). Biodistribution of Radioactively Labeled Splice Modulating Antisense Oligonucleotides After Intracerebroventricular and Intrathecal Injection in Mice. Nucleic Acid Ther..

[53] Ou X., Tan T., He L., Li Y., Li J., Kuang A. (2005). Antitumor effects of radioiodinated antisense oligonuclide mediated by VIP receptor. Cancer Gene Ther..

[54] Younes C.K., Boisgard R., Tavitian B. (2002). Labelled oligonucleotides as radiopharmaceuticals: pitfalls, problems and perspectives. Curr. Pharm. Des..

[55] He C., Migawa M.T., Chen K., Weston T.A., Tanowitz M., Song W., Guagliardo P., Iyer K.S., Bennett C.F., Fong L.G. (2021). High-resolution visualization and quantification of nucleic acid-based therapeutics in cells and tissues using Nanoscale secondary ion mass spectrometry (NanoSIMS). Nucleic Acids Res..

[56] Acosta R., Montañez C., Gómez P., Cisneros B. (2002). Delivery of antisense oligonucleotides to PC12 cells. Neurosci. Res..

[57] Nilsson J.R., Baladi T., Gallud A., Baždarević D., Lemurell M., Esbjörner E.K., Wilhelmsson L.M., Dahlén A. (2021). Fluorescent base analogues in gapmers enable stealth labeling of antisense oligonucleotide therapeutics. Sci. Rep..

[58] Heissig P., Schrimpf W., Hadwiger P., Wagner E., Lamb D.C. (2017). Monitoring integrity and localization of modified single-stranded RNA oligonucleotides using ultrasensitive fluorescence methods. PLoS One.

[59] Buntz A., Killian T., Schmid D., Seul H., Brinkmann U., Ravn J., Lindholm M., Knoetgen H., Haucke V., Mundigl O. (2019). Quantitative fluorescence imaging determines the absolute number of locked nucleic acid oligonucleotides needed for suppression of target gene expression. Nucleic Acids Res..

[60] Derbis M., Kul E., Niewiadomska D., Sekrecki M., Piasecka A., Taylor K., Hukema R.K., Stork O., Sobczak K. (2021). Short antisense oligonucleotides alleviate the pleiotropic toxicity of RNA harboring expanded CGG repeats. Nat. Commun..

[61] Bennett C.F., Chiang M.Y., Chan H., Shoemaker J.E., Mirabelli C.K. (1992). Cationic lipids enhance cellular uptake and activity of phosphorothioate antisense oligonucleotides. Mol. Pharmacol..

[62] Matsuzono K., Imamura K., Murakami N., Tsukita K., Yamamoto T., Izumi Y., Kaji R., Ohta Y., Yamashita T., Abe K., Inoue H. (2017). Antisense Oligonucleotides Reduce RNA Foci in Spinocerebellar Ataxia 36 Patient iPSCs. Mol. Ther. Nucleic Acids.

[63] Wang F., Flanagan J., Su N., Wang L.C., Bui S., Nielson A., Wu X., Vo H.T., Ma X.J., Luo Y. (2012). RNAscope: a novel in situ RNA analysis platform for formalin-fixed, paraffin-embedded tissues. J. Mol. Diagn..

[64] Chimento D.P., Anderson A.L., Fial I., Ascoli C.A. (2025). Bioanalytical Assays for Oligonucleotide Therapeutics: Adding Antibody-Based Immunoassays to the Toolbox as an Orthogonal Approach to LC-MS/MS and Ligand Binding Assays. Nucleic Acid Ther..

[65] Kuijper E.C., van der Graaf L., Pepers B.A., Guimarães Ramos M., Korhorn S., Toonen L.J.A., Cats D., Buijsen R.A.M., Mina E., Mei H., van Roon-Mom W.M.C. (2025). Determining off-target effects of splice-switching antisense oligonucleotides using short read RNAseq in neuronally differentiated human induced pluripotent stem cells. Hum. Mol. Genet..

[66] Hauser S., Helm J., Kraft M., Korneck M., Hübener-Schmid J., Schöls L. (2022). Allele-specific targeting of mutant ataxin-3 by antisense oligonucleotides in SCA3-iPSC-derived neurons. Mol. Ther. Nucleic Acids.

[67] Braatz C., Komes M.P., Ravichandran K.A., de Fragas M.G., Griep A., Schwartz S., McManus R.M., Heneka M.T. (2024). NLRP3-directed antisense oligonucleotides reduce microglial immunoactivities in vitro. J. Neurochem..

[68] Hagedorn P.H., Brown J.M., Easton A., Pierdomenico M., Jones K., Olson R.E., Mercer S.E., Li D., Loy J., Høg A.M. (2022). Acute Neurotoxicity of Antisense Oligonucleotides After Intracerebroventricular Injection Into Mouse Brain Can Be Predicted from Sequence Features. Nucleic Acid Ther..

[69] Jia C., Lei Mon S.S., Yang Y., Katsuyama M., Yoshida-Tanaka K., Nagata T., Yoshioka K., Yokota T. (2023). Change of intracellular calcium level causes acute neurotoxicity by antisense oligonucleotides via CSF route. Mol. Ther. Nucleic Acids.

[70] Miller R., Paquette J., Barker A., Sapp E., McHugh N., Bramato B., Yamada N., Alterman J., Echeveria D., Yamada K. (2024). Preventing acute neurotoxicity of CNS therapeutic oligonucleotides with the addition of Ca2+ and Mg2+ in the formulation. Mol. Ther. Nucleic Acids.

[71] Lindow M., Vornlocher H.P., Riley D., Kornbrust D.J., Burchard J., Whiteley L.O., Kamens J., Thompson J.D., Nochur S., Younis H. (2018). Assessing unintended hybridization-induced biological effects of oligonucleotides. Nat. Biotechnol..

[72] Varani G., McClain W.H. (2000). The G x U wobble base pair. A fundamental building block of RNA structure crucial to RNA function in diverse biological systems. EMBO Rep..

[73] Hwang G., Kwon M., Seo D., Kim D.H., Lee D., Lee K., Kim E., Kang M., Ryu J.H. (2024). ASOptimizer: Optimizing antisense oligonucleotides through deep learning for IDO1 gene regulation. Mol. Ther. Nucleic Acids.

[74] Yoshida T., Naito Y., Sasaki K., Uchida E., Sato Y., Naito M., Kawanishi T., Obika S., Inoue T. (2018). Estimated number of off-target candidate sites for antisense oligonucleotides in human mRNA sequences. Genes Cells.

[75] Leckie J., Wu S., Standell T., Yokota T. (2025). Artificial Intelligence-Driven Design of Antisense Oligonucleotides for Precision Medicine in Neuromuscular Disorders. Genes.

[76] Kimi J., Korczak P., Vialet B., Roubin E., Barthélémy P., Campagne S., Malard F. (2025). ASOG: AntiSense Oligonucleotide Generator. Comput. Struct. Biotechnol. J..

[77] Linninger A.A., Barua D., Hang Y., Iadevaia S., Vakilynejad M. (2023). A mechanistic pharmacokinetic model for intrathecal administration of antisense oligonucleotides. Front. Physiol..

[78] Johansson K., Maouia A., Rebetz J., Marcoux G., Shannon O., Italiano J.E., Narayanan P., Henry S., Shen L., Semple J.W. (2024). CpG oligonucleotides induce acute murine thrombocytopenia dependent on toll-like receptor 9 and spleen tyrosine kinase pathways. J. Thromb. Haemost..

[79] van Leeuwen D., Cipullo M., Williams E.C., Iannetta A., Chu B., Wang J., Hamza G., Crysnanto D., Guzzi N., Tan J.Y. (2026). Integrating transcriptomics and proteomics to assess antisense oligonucleotide safety and efficacy: a time-resolved approach. NAR Mol. Med..

[80] Pollak A.J., Zhao L., Vickers T.A., Huggins I.J., Liang X.H., Crooke S.T. (2022). Insights into innate immune activation via PS-ASO-protein-TLR9 interactions. Nucleic Acids Res..

[81] Szeky B., Jurakova V., Fouskova E., Feher A., Zana M., Karl V.R., Farkas J., Bodi-Jakus M., Zapletalova M., Pandey S. (2024). Efficient derivation of functional astrocytes from human induced pluripotent stem cells (hiPSCs). PLoS One.

[82] Srinivasan B., Lloyd M.D. (2024). Dose–Response Curves and the Determination of IC50 and EC50 Values Bharath Srinivasan. J. Med. Chem..

[83] Nakayama T., El Achkar C.M., Burbano L.E., Quraishi I.H., Wu J., Li M., Asami Y., Golinski S.R., Sherrill E., Goodlett B.D. (2026). Antisense oligonucleotide-mediated knockdown therapy in two infants with severe KCNT1 epileptic encephalopathy. Nat. Med..

[84] Li D., Mastaglia F.L., Yau W.Y., Chen S., Wilton S.D., Akkari P.A. (2022). Targeted Molecular Therapeutics for Parkinson's Disease: A Role for Antisense Oligonucleotides?. Mov. Disord..

[85] Hamad A.A. (2025). Tofersen for Amyotrophic Lateral Sclerosis: Genetic Treatment With Precision Medicine: The Future of ALS Treatment. J. Clin. Neuromuscul. Dis..

[86] Hamad A.A., Alkhawaldeh I.M., Nashwan A.J., Meshref M., Imam Y. (2025). Tofersen for SOD1 amyotrophic lateral sclerosis: a systematic review and meta-analysis. Neurol. Sci..

